# Multiple-region grey matter atrophy as a predictor for the development of dementia in a community: the Hisayama Study

**DOI:** 10.1136/jnnp-2021-326611

**Published:** 2021-10-20

**Authors:** Taro Nakazawa, Tomoyuki Ohara, Naoki Hirabayashi, Yoshihiko Furuta, Jun Hata, Mao Shibata, Takanori Honda, Takanari Kitazono, Tomohiro Nakao, Toshiharu Ninomiya

**Affiliations:** 1 Department of Neuropsychiatry, Graduate School of Medical Sciences, Kyushu University, Fukuoka, Japan; 2 Department of Epidemiology and Public Health, Graduate School of Medical Sciences, Kyushu University, Fukuoka, Japan; 3 Department of Psychosomatic Medicine, Graduate School of Medical Sciences, Kyushu University, Fukuoka, Japan; 4 Department of Medicine and Clinical Science, Graduate School of Medical Sciences, Kyushu University, Fukuoka, Japan; 5 Department of Center for Cohort Studies, Graduate School of Medical Sciences, Kyushu University, Fukuoka, Japan

**Keywords:** dementia, alzheimer's disease, vascular dementia, neuropsychiatry, epidemiology

## Abstract

**Objective:**

To assess the association of regional grey matter atrophy with dementia risk in a general older Japanese population.

**Methods:**

We followed 1158 dementia-free Japanese residents aged ≥65 years for 5.0 years. Regional grey matter volume (GMV) at baseline was estimated by applying voxel-based morphometry methods. The GMV-to-total brain volume ratio (GMV/TBV) was calculated, and its association with dementia risk was estimated using Cox proportional hazard models. We assessed whether the predictive ability of a model based on known dementia risk factors could be improved by adding the total number of regions with grey matter atrophy among dementia-related brain regions, where the cut-off value for grey matter atrophy in each region was determined by receiver operating characteristic curves.

**Results:**

During the follow-up, 113 participants developed all-cause dementia, including 83 with Alzheimer’s disease (AD). Lower GMV/TBV of the medial temporal lobe, insula, hippocampus and amygdala were significantly/marginally associated with higher risk of all-cause dementia and AD (all p for trend ≤0.08). The risks of all-cause dementia and AD increased significantly with increasing total number of brain regions exhibiting grey matter atrophy (both p for trend <0.01). Adding the total number of regions with grey matter atrophy into a model consisting of known risk factors significantly improved the predictive ability for AD (Harrell’s c-statistics: 0.765–0.802; p=0.02).

**Conclusions:**

Our findings suggest that the total number of regions with grey matter atrophy among the medial temporal lobe, insula, hippocampus and amygdala is a significant predictor for developing dementia, especially AD, in the general older population.

## Introduction

Brain atrophy is one of the morphological features of dementia.^1^ Generally, brain atrophy progresses with ageing.^2–4^ Clinical and neuropathological studies have revealed that regions of grey matter atrophy vary by the disease^5^ and patients with dementia or mild cognitive impairment (MCI) have remarkable grey matter atrophy in several brain regions, including the hippocampus.^1 6–8^ Other studies have shown that brain atrophy occurs before cognitive impairment becomes apparent.^9 10^ These findings suggest that identifying the dementia-related brain regions may be useful for risk assessment of incident dementia.

Several population-based prospective studies have examined the association between atrophy in specific brain regions, such as the hippocampus, as well as in the whole brain and risk of dementia.^11–15^ In addition, clinical and neuropathological studies have also suggested that grey matter atrophy in multiple specific brain regions—that is, more than one or two specific regions—could be involved in the onset of dementia, and the regions of grey matter atrophy in the development of dementia differ among individuals.^16 17^ Therefore, it would be useful to clarify the specific brain regions related to the development of dementia in order to improve risk assessment for the future onset of dementia. Nevertheless, relatively few studies have examined the influence of grey matter atrophy in multiple dementia-related brain regions on the risk assessment for dementia, as well as the prediction ability for incident dementia.^7 8 15^

This study aimed to identify specific brain regions in which grey matter atrophy is associated with the development of dementia using brain MRI data and prospective cohort data for dementia in a general older population of Japanese. In addition, we also investigated the influence of the total number of regions with grey matter atrophy among dementia-related brain regions on the ability to predict future onset of dementia.

## Methods

### Study population

In the Hisayama Study, which is an ongoing population-based longitudinal study of cerebro-cardiovascular diseases that began in 1961 in the town of Hisayama, a full-community survey for dementia has been repeated every 5–7 years since 1985.^18^ Among 2036 residents aged 65 years and older in this town, a total of 1906 residents (1126 women and 780 men) (participation rate: 93.6%) participated in the examination for cognitive function and health status in the years 2012–2013. Among them, 1342 participants underwent brain MRI scanning for this study. After excluding 151 participants who had dementia at baseline, 1 participant who did not complete the examination for cognitive function at baseline, and 32 participants without available MRI data (20 without T1-weighted 3-dimensional images, 5 without fluid attenuated inversion recovery (FLAIR) images, 4 with metal artefacts, 2 with excessive motion artefact and 1 who did not consent to use the MRI data), the remaining 1158 participants (646 women and 512 men) were enrolled in this study. We obtained written informed consent from all the participants.

### Follow-up surveys

The participants were followed for a median of 5.0 years (IQR 4.9–5.1 years) from the baseline examination. As reported previously,^18^ we established a daily monitoring system comprising the study team, local physicians, and members of the town’s Health and Welfare Office to regularly collect information on new neurologic events, including dementia and stroke. Regular health examinations were performed annually to identify incident dementia cases. For participants who did not undergo regular health examinations or moved out of town, we performed postal and telephone surveys. Moreover, to precisely detect dementia cases to the greatest extent possible, we conducted comprehensive neuropsychological screening for dementia in 2017–2018, which 1017 participants (87.8% of total participants) underwent. A total of 141 participants who did not participate in the neuropsychological screening in 2017–2018 were evaluated for potential dementia by collecting all the available information and/or direct interview by expert psychiatrists. Once dementia or any neurological symptoms including cognitive decline were suspected, a psychiatrist and stroke physician from the study team carefully evaluated the participant for the presence or absence of dementia. When a participant died, we conducted comprehensive investigations, including interviews of the family or attending physician and a review of all the available clinical records, including neuroimaging (CT/MRI). The participants were followed up until the date of neuropsychological screening for dementia in 2017–2018 or 31 March 2018 for those who did not participate in the neuropsychological screening in 2017–2018. No participants were lost to follow-up except for deceased cases.

### Diagnosis of dementia

The diagnoses of dementia and MCI were made using the criteria of the Diagnostic and Statistical Manual of Mental Disorders, Third Edition, Revised,^19^ and the clinical criteria reported by Petersen *et al*,^20^ respectively. Participants diagnosed as having Alzheimer’s disease (AD) met the diagnostic criteria of the National Institute of Neurological and Communicative Disorders and Stroke and the AD and Related Disorders Association,^21^ and participants diagnosed with vascular dementia (VaD) met the criteria of the National Institute of Neurological Disorders and Stroke–Association Internationale pour la Recherche et I’Enseignement en Neurosciences.^22^ In the screening survey, we used the Mini-Mental State Examination (MMSE).^23^ For participants who were suspected of having dementia or MCI, comprehensive investigations including the Wechsler Memory Scale of logical memory^24^ were carried out by expert psychiatrists. We defined MCI as either of: (1) objective cognitive impairment based on the neuropsychological data; or (2) any cognitive complaint by a family member, the town’s Health and Welfare Office members or local practitioners in individuals who showed no evidence of dementia. Expert psychiatrists and stroke physicians in the study team adjudicated every case of dementia and MCI together.

### MRI analysis

Detailed information on the MRI analysis is provided in online supplemental material. Briefly, using a 1.5-Tesla MRI scanner (Intera Pulsar; Philips Medical Systems, Best, the Netherlands) with a multichannel head coil, we examined 3-dimensional T1-weighted images, conventional T1- and T2-weighted images, FLAIR, T2*-weighted images, and magnetic resonance angiographic images of the brain. The 3-dimensional T1-weighted images were converted to Neuroimaging Informatics Technology Initiative format and then segmented into three components (grey matter, white matter and cerebrospinal fluid) by using VBM8 Toolbox version 435 (University of Jena, Germany) in SPM8 (University College London, UK) running in MATLAB (The Mathworks, Natick, Massachusetts, USA). The International Consortium for Brain Mapping template for East Asian brains was used for anatomical setting. Since white matter hyperintensities (WMH) were often misclassified as grey matter, we corrected white matter and grey matter images by using binarised WMH masks. Segmented grey matter images were normalised and modulated to compensate for the volumetric effects of expansion/shrinking in spatial normalisation. Based on the preceding cortical parcellation, grey matter volume (GMV) of the frontal, temporal, medial temporal (including entorhinal and parahippocampus), parietal, occipital and insular lobes and the cingulate, hippocampus, accumbens, amygdala, caudate, pallidum, putamen and thalamus were computed using the Neuromorphometrics atlas of SPM12 (Neuromorphometrics, Somerville, Massachusetts, USA). The total brain volume (TBV) was calculated as the sum of the grey matter and white matter volumes. The intracranial volume (ICV) was calculated as the sum of the TBV and the cerebrospinal fluid volumes. We calculated the TBV to ICV ratio (TBV/ICV) as an indicator of global brain atrophy. As an indicator of regional grey matter atrophy beyond total brain atrophy, the GMV to TBV ratio (GMV/TBV) for each brain region was calculated. Cerebrovascular lesions were defined as brain infarction or haemorrhage on MRI regardless of the presence or absence of neurological symptoms.

10.1136/jnnp-2021-326611.supp1 Supplementary data

### Risk-factor measurements

In the baseline survey, we obtained the information on education, medication, and lifestyle factors, measured blood pressure, plasma glucose, serum total cholesterol, body weight and height, and took an ECG. Detailed information on the risk factors is provided in online supplemental material.

### Statistical analysis

Details of the statistical analysis are also given in online supplemental material. Briefly, the HRs and their 95% CIs of the quartiles of TBV/ICV or the quartiles of GMV/TBV in each brain region for the development of dementia and its subtypes were computed by using a Cox proportional hazards model. False discovery rate (FDR) correction^25^ was performed to verify the multiple comparisons for which a significance level with a q-value of FDR correction was defined as <0.10.^26^ We assessed the association between the total number of regions exhibiting grey matter atrophy among four dementia-related brain regions, where the cut-off value for grey matter atrophy in each region was determined by receiver operating characteristic (ROC) curves,^27^ and the risk of dementia. We also assessed whether the predictive ability of a model based on known dementia risk factors could be improved by adding the hippocampal atrophy or total number of regions with grey matter atrophy among dementia-related brain regions.

## Results

Table 1 shows the baseline characteristics of the total study population and the age-adjusted and sex-adjusted mean values or frequencies of potential risk factors for dementia according to the quartiles of TBV/ICV. The mean values of age and frequencies of male gender, antihypertensive medication, diabetes mellitus and MCI decreased significantly with higher TBV/ICV levels. For Spearman’s correlation coefficients between TBV/ICV and each risk factor, a negative association was observed for the mean value of age and the frequencies of male gender, antihypertensive medication, hypertension, diabetes mellitus and smoking habits (online supplemental table s-1).

**Table 1:** Baseline characteristics of the total study population and age-adjusted and sex-adjusted mean values or frequencies of potential risk factors for dementia according to quartiles of the total brain volume TBV-to-ICV ratio at baseline

Risk factors at baseline	Total population (n=1158)	TBV/ICV (%)				P for trend
		Q1 (71.54–76.81) (n=289)	Q2 (76.82–78.43) (n=290)	Q3 (78.44–79.80) (n=290)	Q4 (79.81–85.48) (n=289)	
Age, years	73.6 (6.2)†	78.7 (0.3)‡	74.9 (0.3)‡	71.6 (0.3)‡	69.4 (0.3)‡	<0.001
Male sex, %	44.2	69.0§	50.0§	37.0§	21.8§	<0.001
Education ≤9 years, %	35.5	33.3§	35.8§	35.5§	35.5§	0.70
Systolic blood pressure, mm Hg	134.0 (18.4)†	134.5 (1.2)‡	133.7 (1.1)‡	134.0 (1.1)‡	133.9 (1.2)‡	0.97
Diastolic blood pressure, mm Hg	76.4 (10.9)†	75.9 (0.7)‡	76.0 (0.6)‡	77.3 (0.6)‡	76.5 (0.7)‡	0.45
Antihypertensive medication, %	54.9	59.4§	59.8§	52.1§	49.5§	0.02
Hypertension, %	69.7	74.1§	73.0§	67.4§	67.2§	0.08
Diabetes mellitus, %	23.6	34.6§	26.7§	18.8§	13.6§	<0.001
Serum total cholesterol, mmol/L	198.1 (35.6)†	194.7 (2.2)‡	197.1 (2.0)‡	199.8 (2.0)‡	200.7 (2.2)‡	0.29
Body mass index, kg/m^2^	23.2 (3.3)†	23.0 (0.2)‡	23.3 (0.2)‡	23.4 (0.2)‡	23.0 (0.2)‡	0.21
ECG abnormalities, %	16.3	19.0§	13.3§	15.8§	13.8§	0.26
Cerebrovascular lesions, %	34.7	37.8§	35.0§	33.9§	28.6§	0.07
Smoking habits, %	8.9	7.4§	6.4§	6.2§	3.7§	0.08
Alcohol intake, %	42.9	42.4§	46.4§	37.6§	40.8§	0.44
Regular exercise, %	20.4	18.5§	25.6§	17.2§	19.9§	0.71
MMSE <24, %	5.7	7.4§	6.3§	4.7§	3.8§	0.09
Mild cognitive impairment, %	13.6	14.4§	15.7§	13.4§	6.8§	0.03

Age was adjusted for sex. Sex was adjusted for age.

Hypertension was defined as blood pressure ≥140/90 mm Hg and/or current use of antihypertensive medication. ECG abnormalities were defined as Minnesota code 3–1, 4–1, 4–2, 4–3 or 8–3. Regular exercise was defined as engaging in any form of physical exercise three or more times a week during leisure time. Cerebrovascular lesions were defined as brain infarction or haemorrhage on MRI regardless of the presence of absence of neurological symptoms. Smoking habits and alcohol intake were classified as current use or not.

†Values are shown as mean (SD).

‡Values are shown as mean (SE) after adjustment for age and sex.

§Frequencies were adjusted for age and sex.

GMV, grey matter volume; ICV, intracranial volume; MMSE, Mini–Mental Statement Examination; TBV, total brain volume.

During the follow-up period, 113 participants (47 men and 66 women) developed all-cause dementia. Among them, one participant had a mixed type of AD and VaD, and this case was counted as an event in the analysis for each subtype. In all, 83 participants developed AD, and 14 participants developed VaD.

Table 2 shows the estimated risk of all-cause dementia and its subtypes according to TBV/ICV levels. The age- and sex-adjusted risk of all-cause dementia increased significantly with lower TBV/ICV levels (p for trend=0.002). These associations were not changed after adjustment for age, sex, education status, systolic blood pressure, antihypertensive medication, diabetes mellitus, serum total cholesterol, body mass index, ECG abnormalities, cerebrovascular lesions on MRI, smoking habits, alcohol intake and regular exercise (*p* for trend=0.003). Subjects in the first quartile of TBV/ICV had a 3.36 (95% CI 1.48 to 7.65) times greater risk of all-cause dementia than those in the fourth quartile. With regard to subtypes of dementia, the multivariable-adjusted risk of AD increased significantly with lower quartile of TBV/ICV (p for trend=0.01), but there was no evidence of significant association between TBV/ICV and the risk of VaD (p for trend=0.47)

**Table 2:** Adjusted HRs (95% CI) of all-cause dementia and its subtypes according to quartiles of the TBV-to-ICV ratio

TBV/ICV (%)	No of subjects	No of events	HR (95% CI)	
			Model 1†	Model 2‡
All-cause dementia				
Q1 (71.54–76.81)	289	51	3.60 (1.59 to 8.14)*	3.36 (1.48–7.65)*
Q2 (76.82–78.43)	290	33	2.41 (1.07 to 5.41)*	2.31 (1.02–5.20)*
Q3 (78.44–79.80)	290	21	2.28 (1.001 to 5.20)*	2.10 (0.92–4.84)
Q4 (79.81–85.48)	289	8	1.00 (Reference)	1.00 (Reference)
			P for trend=0.002	P for trend=0.003
Alzheimer’s disease				
Q1 (71.54–76.81)	289	37	4.20 (1.53 to 11.53)*	4.09 (1.48–11.31)*
Q2 (76.82–78.43)	290	24	2.72 (0.996 to 7.41)	2.66 (0.97–7.31)
Q3 (78.44–79.80)	290	17	2.93 (1.07 to 8.02)*	2.70 (0.98–7.50)
Q4 (79.81–85.48)	289	5	1.00 (Reference)	1.00 (Reference)
			P for trend=0.01	P for trend=0.01
Vascular dementia				
Q1 (71.54–76.81)	289	6	1.86 (0.31 to 11.34)	1.54 (0.26–9.29)
Q2 (76.82–78.43)	290	4	1.25 (0.21 to 7.34)	1.16 (0.20–6.83)
Q3 (78.44–79.80)	290	2	0.81 (0.11 to 5.81)	0.72 (0.10–5.32)
Q4 (79.81–85.48)	289	2	1.00 (Reference)	1.00 (Reference)
			P for trend=0.37	P for trend=0.47

*P<0.05 vs Q4.

†Model 1: adjusted for age and sex.

‡Model 2: adjusted for age, sex, education status, systolic blood pressure, antihypertensive medication, diabetes mellitus, serum total cholesterol, body mass index, ECG abnormalities, cerebrovascular lesions on MRI, smoking habits, alcohol intake and regular exercise.

ICV, intracranial volume; TBV, total brain volume.

Next, we investigated the association of GMV/TBV levels for each brain region, as an indicator of regional grey matter atrophy beyond total brain atrophy, with the development of dementia. Spearman’s correlation coefficients for the associations of GMV/TBV for each brain lobe or region with each risk factor are shown in online supplemental tables s-1 and s-2. Tables 3 and 4 show the association between the risk of all-cause dementia and the GMV/TBV levels for each brain lobe, and the basal ganglia, limbic system, and thalamus. The risk of all-cause dementia increased significantly with lower GMV/TBV of the medial temporal lobe, insula, hippocampus, and amygdala after adjustment for the above-mentioned risk factors plus TBV/ICV (all p for trend ≤0.02 and q-values of FDR correction ≤0.06) (online supplemental tables s-3). The multivariable-adjusted risk of all-cause dementia increased significantly in participants in the first quartile of GMV/TBV of the medial temporal lobe (HR 1.80, 95% CI 1.07 to 3.00), insula (HR 1.80, 95% CI 1.02 to 3.17), hippocampus (HR 3.21, 95% CI 1.61 to 6.40) and amygdala (HR 2.06, 95% CI 1.12 to 3.78) compared with those in the fourth quartile. No significant associations were observed for GMV/TBV of the frontal, temporal, parietal and occipital lobes, or other regions of the basal ganglia, limbic system and thalamus. The sensitivity analyses after excluding subjects with MCI, those with an MMSE score of <24, or those who developed dementia within 1 year did not change the present findings substantially (online supplemental tables s-4–s-6). With regard to dementia subtypes, lower GMV/TBV levels of four dementia-related brain regions (ie, the medial temporal lobe, insula, hippocampus and amygdala) were significantly or marginally associated with a greater risk of AD after adjusting for the above-mentioned risk factors plus TBV/ICV (all p for trend ≤0.08) (table 5). On the other hand, there were no clear associations for VaD.

**Table 3:** Adjusted HRs (95% CI) of all-cause dementia according to quartiles of the GMV-to-TBV ratio of each brain lobe

	No of subjects	No of events	HR (95% CI)		
			Model 1†	Model 2‡	Model 3§
Frontal GMV/TBV (%)					
Q1 (8.19–12.00)	289	46	1.26 (0.72 to 2.22)	1.15 (0.65 to 2.04)	0.96 (0.54 to 1.73)
Q2 (12.01–12.74)	290	25	0.92 (0.51 to 1.69)	0.90 (0.49 to 1.65)	0.87 (0.47 to 1.61)
Q3 (12.75–13.37)	290	22	0.95 (0.52 to 1.75)	0.92 (0.50 to 1.72)	0.90 (0.49 to 1.68)
Q4 (13.38–15.80)	289	20	1.00 (Reference)	1.00 (Reference)	1.00 (Reference)
			P for trend=0.34	P for trend=0.53	P for trend=0.96
Temporal GMV/TBV (%)					
Q1 (5.80–8.79)	289	44	1.54 (0.84 to 2.85)	1.53 (0.82 to 2.85)	1.46 (0.79 to 2.70)
Q2 (8.80–9.33)	290	36	1.88 (1.02 to 3.46)*	1.71 (0.92 to 3.17)	1.78 (0.96 to 3.31)
Q3 (9.34–9.77)	290	18	1.03 (0.52 to 2.06)	1.01 (0.51 to 2.02)	1.08 (0.54 to 2.16)
Q4 (9.78–10.96)	289	15	1.00 (Reference)	1.00 (Reference)	1.00 (Reference)
			P for trend=0.07	P for trend=0.08	P for trend=0.14
Medial temporal GMV/TBV (%)					
Q1 (0.40–0.79)	289	51	1.72 (1.03 to 2.86)*	1.72 (1.03 to 2.87)*	1.80 (1.07 to 3.00)*
Q2 (0.80–0.84)	290	19	0.75 (0.41 to 1.39)	0.76 (0.41 to 1.42)	0.84 (0.45 to 1.57)
Q3 (0.85–0.89)	290	21	0.93 (0.51 to 1.69)	0.87 (0.47 to 1.60)	0.97 (0.52 to 1.80)
Q4 (0.90–1.09)	289	22	1.00 (Reference)	1.00 (Reference)	1.00 (Reference)
			P for trend=0.02	P for trend=0.02	P for trend=0.02
Parietal GMV/TBV (%)					
Q1 (4.91–7.46)	289	25	0.60 (0.34 to 1.05)	0.58 (0.33 to 1.03)	0.56 (0.31 to 0.99)*
Q2 (7.47–7.84)	290	27	0.78 (0.45 to 1.33)	0.69 (0.40 to 1.21)	0.72 (0.41 to 1.26)
Q3 (7.85–8.20)	290	31	1.06 (0.64 to 1.75)	1.10 (0.65 to 1.85)	1.11 (0.65 to 1.88)
Q4 (8.21–11.1)	289	30	1.00 (Reference)	1.00 (Reference)	1.00 (Reference)
			P for trend=0.04	P for trend=0.09	P for trend=0.06
Occipital GMV/TBV (%)					
Q1 (4.35–5.54)	289	28	0.85 (0.51 to 1.42)	0.87 (0.51 to 1.48)	0.85 (0.49 to 1.45)
Q2 (5.55–5.85)	290	26	0.87 (0.52 to 1.47)	0.81 (0.48 to 1.37)	0.84 (0.49 to 1.43)
Q3 (5.86–6.16)	290	28	0.92 (0.55 to 1.54)	0.91 (0.54 to 1.54)	1.01 (0.60 to 1.72)
Q4 (6.17–7.17)	289	31	1.00 (Reference)	1.00 (Reference)	1.00 (Reference)
			P for trend=0.51	P for trend=0.52	P for trend=0.43
Insular GMV/TBV (%)					
Q1 (0.61–1.03)	289	49	1.90 (1.10 to 3.29)*	1.91 (1.09 to 3.35)*	1.80 (1.02 to 3.17)*
Q2 (1.04–1.10)	290	28	1.28 (0.71 to 2.32)	1.26 (0.69 to 2.30)	1.26 (0.69 to 2.32)
Q3 (1.11–1.17)	290	17	0.72 (0.37 to 1.40)	0.73 (0.38 to 1.43)	0.72 (0.37 to 1.42)
Q4 (1.18–1.46)	289	19	1.00 (Reference)	1.00 (Reference)	1.00 (Reference)
			P for trend=0.002	P for trend=0.002	P for trend=0.004

*P<0.05 vs Q4.

†Model 1: adjusted for age and sex.

‡Model 2: adjusted for age, sex, education status, systolic blood pressure, antihypertensive medication, diabetes mellitus, serum total cholesterol, body mass index, ECG abnormalities, cerebrovascular lesions on MRI, smoking habits, alcohol intake and regular exercise.

§Model 3: adjusted for the variables in model two plus TBV/ICV.

GMV, grey matter volume; ICV, intracranial volume; TBV, total brain volume.

**Table 4:** Adjusted HRs (95% CI) of all-cause dementia according to quartiles of the GMV-to-TMV ratio of the basal ganglia, limbic system, and thalamus

	No of subjects	No of events	HR (95% CI)		
			Model 1†	Model 2‡	Model 3§
Cingulate GMV/TBV (%)					
Q1 (1.63–2.18)	289	36	1.37 (0.76 to 2.46)	1.35 (0.75 to 2.43)	1.29 (0.72 to 2.34)
Q2 (2.19–2.28)	290	35	1.50 (0.84 to 2.68)	1.54 (0.86 to 2.76)	1.54 (0.86 to 2.77)
Q3 (2.29–2.37)	290	24	1.09 (0.59 to 2.02)	1.17 (0.63 to 2.19)	1.23 (0.66 to 2.30)
Q4 (2.38–2.72)	289	18	1.00 (Reference)	1.00 (Reference)	1.00 (Reference)
			P for trend=0.19	P for trend=0.26	P for trend=0.36
Hippocampal GMV/TBV (%)					
Q1 (0.45–0.77)	289	56	3.42 (1.73 to 6.76)**	3.62 (1.82 to 7.20)**	3.21 (1.61 to 6.40)**
Q2 (0.78–0.81)	290	27	1.92 (0.94 to 3.93)	2.07 (1.01 to 4.24)*	2.07 (1.01 to 4.26)*
Q3 (0.82–0.86)	290	19	1.51 (0.71 to 3.20)	1.51 (0.71 to 3.22)	1.44 (0.67 to 3.09)
Q4 (0.87–1.01)	289	11	1.00 (Reference)	1.00 (Reference)	1.00 (Reference)
			P for trend <0.001	P for trend <0.001	P for trend <0.001
Accumbens GMV/TBV (%)					
Q1 (0.04–0.08)	289	52	2.69 (1.30 to 5.56)*	2.61 (1.26 to 5.37)*	2.11 (1.01 to 4.39)*
Q2 (0.09–0.09)	290	28	1.66 (0.79 to 3.52)	1.56 (0.74 to 3.31)	1.40 (0.66 to 2.98)
Q3 (0.10–0.10)	290	23	1.83 (0.86 to 3.87)	1.87 (0.88 to 3.98)	1.79 (0.84 to 3.81)
Q4 (0.11–0.14)	289	10	1.00 (Reference)	1.00 (Reference)	1.00 (Reference)
			P for trend=0.01	P for trend=0.01	P for trend=0.08
Amygdala GMV/TBV (%)					
Q1 (0.11–0.21)	289	57	2.14 (1.16 to 3.93)*	2.24 (1.22 to 4.13)*	2.06 (1.12 to 3.78)*
Q2 (0.22–0.23)	290	26	1.40 (0.74 to 2.67)	1.45 (0.76 to 2.77)	1.49 (0.78 to 2.84)
Q3 (0.24–0.24)	290	15	0.91 (0.44 to 1.86)	0.87 (0.42 to 1.82)	0.88 (0.42 to 1.83)
Q4 (0.25–0.31)	289	15	1.00 (Reference)	1.00 (Reference)	1.00 (Reference)
			P for trend=0.002	P for trend=0.001	P for trend=0.003
Caudate GMV/TBV (%)					
Q1 (0.16–0.31)	289	44	1.55 (0.81 to 2.98)	1.47 (0.77 to 2.83)	0.98 (0.49 to 1.93)
Q2 (0.32–0.37)	290	34	1.56 (0.82 to 2.97)	1.50 (0.79 to 2.88)	1.20 (0.62 to 2.32)
Q3 (0.38–0.45)	290	21	1.17 (0.59 to 2.31)	1.14 (0.58 to 2.26)	1.04 (0.52 to 2.06)
Q4 (0.46–0.86)	289	14	1.00 (Reference)	1.00 (Reference)	1.00 (Reference)
			P for trend=0.14	P for trend=0.19	P for trend=0.93
Pallidum GMV/TBV (%)					
Q1 (0.01–0.01)	289	36	0.89 (0.56 to 1.42)	1.01 (0.62 to 1.64)	1.02 (0.62 to 1.66)
Q2 (0.01–0.02)	290	20	0.63 (0.36 to 1.09)	0.70 (0.40 to 1.23)	0.75 (0.42 to 1.32)
Q3 (0.02–0.02)	290	22	0.74 (0.43 to 1.27)	0.81 (0.47 to 1.40)	0.81 (0.47 to 1.41)
Q4 (0.03–0.08)	289	35	1.00 (Reference)	1.00 (Reference)	1.00 (Reference)
			P for trend=0.56	P for trend=0.93	P for trend=0.99
Putamen GMV/TBV (%)					
Q1 (0.08–0.33)	289	46	1.19 (0.73 to 1.96)	1.34 (0.81 to 2.22)	1.10 (0.66 to 1.84)
Q2 (0.34–0.43)	290	20	0.71 (0.40 to 1.28)	0.73 (0.40 to 1.35)	0.69 (0.37 to 1.27)
Q3 (0.44–0.51)	290	20	0.77 (0.43 to 1.37)	0.81 (0.45 to 1.46)	0.77 (0.43 to 1.38)
Q4 (0.52–1.04)	289	27	1.00 (Reference)	1.00 (Reference)	1.00 (Reference)
			P for trend=0.42	P for trend=0.22	P for trend=0.63
Thalamus GMV/TBV (%)					
Q1 (0.40–0.64)	289	48	1.49 (0.85 to 2.62)	1.60 (0.90 to 2.85)	1.19 (0.66 to 2.16)
Q2 (0.65–0.72)	290	26	1.09 (0.60 to 2.00)	1.07 (0.58 to 1.98)	0.98 (0.53 to 1.82)
Q3 (0.73–0.79)	290	20	0.95 (0.51 to 1.79)	0.96 (0.51 to 1.82)	0.91 (0.48 to 1.74)
Q4 (0.80–1.15)	289	19	1.00 (Reference)	1.00 (Reference)	1.00 (Reference)
			P for trend=0.10	P for trend=0.06	P for trend=0.46

*P<0.05 vs Q4, **p<0.001 vs Q4.

†Model 1: adjusted for age and sex.

‡Model 2: adjusted for age, sex, education status, systolic blood pressure, antihypertensive medication, diabetes mellitus, serum total cholesterol, body mass index, ECG abnormalities, cerebrovascular lesions on MRI, smoking habits, alcohol intake and regular exercise.

§Model 3: adjusted for the variables in model two plus TBV/ICV.

GMV, grey matter volume; ICV, intracranial volume; TBV, total brain volume.

**Table 5:** Multivariable-adjusted HRs (95% CI) of dementia subtypes according to quartiles of the GMV-to-TBV ratio of the medial temporal lobe, insula, hippocampus and amygdala

	No of subjects	Alzheimer’s disease		Vascular dementia	
		No of events	Multivariable-adjusted HR (95% CI)	No of events	Multivariable-adjusted HR (95% CI)
Medial temporal GMV/TBV (%)					
Q1 (0.40–0.79)	289	36	1.51 (0.84 to 2.70)	4	1.30 (0.28 to 6.04)
Q2 (0.80–0.84)	290	16	0.84 (0.43 to 1.67)	2	0.86 (0.14 to 5.30)
Q3 (0.85–0.89)	290	13	0.69 (0.33 to 1.45)	5	1.96 (0.44 to 8.67)
Q4 (0.90–1.09)	289	18	1.00 (Reference)	3	1.00 (Reference)
			P for trend=0.08		P for trend=0.99
Insular GMV/TBV (%)					
Q1 (0.61–1.03)	289	35	1.88 (0.96 to 3.66)	6	1.73 (0.40 to 7.49)
Q2 (1.04–1.10)	290	22	1.42 (0.70 to 2.87)	3	1.21 (0.23 to 6.31)
Q3 (1.11–1.17)	290	12	0.72 (0.33 to 1.60)	2	0.60 (0.10 to 3.78)
Q4 (1.18–1.46)	289	14	1.00 (Reference)	3	1.00 (Reference)
			P for trend=0.01		P for trend=0.30
Hippocampal GMV/TBV (%)					
Q1 (0.45–0.77)	289	40	3.57 (1.53 to 8.34)*	6	1.60 (0.33 to 7.74)
Q2 (0.78–0.81)	290	22	2.62 (1.10 to 6.26)*	1	0.28 (0.03 to 2.90)
Q3 (0.82–0.86)	290	14	1.55 (0.61 to 3.94)	4	1.76 (0.36 to 8.46)
Q4 (0.87–1.01)	289	7	1.00 (Reference)	3	1.00 (Reference)
			P for trend <0.001		P for trend=0.86
Amygdala GMV/TBV (%)					
Q1 (0.11–0.21)	289	44	2.41 (1.16 to 5.01)*	4	0.80 (0.18 to 3.62)
Q2 (0.22–0.23)	290	18	1.57 (0.72 to 3.44)	3	0.89 (0.19 to 4.13)
Q3 (0.24–0.24)	290	11	0.94 (0.39 to 2.29)	3	1.18 (0.26 to 5.46)
Q4 (0.25–0.31)	289	10	1.00 (Reference)	4	1.00 (Reference)
			P for trend=0.003		P for trend=0.73

Multivariate adjustment was made for age, sex, education status, systolic blood pressure, antihypertensive medication, diabetes mellitus, serum total cholesterol, body mass index, ECG abnormalities, cerebrovascular lesions on MRI, smoking habits, alcohol intake, regular exercise and TBV/ICV.

*P<0.05 vs Q4.

GMV, grey matter volume; ICV, intracranial volume; TBV, total brain volume.

**Figure 1:**
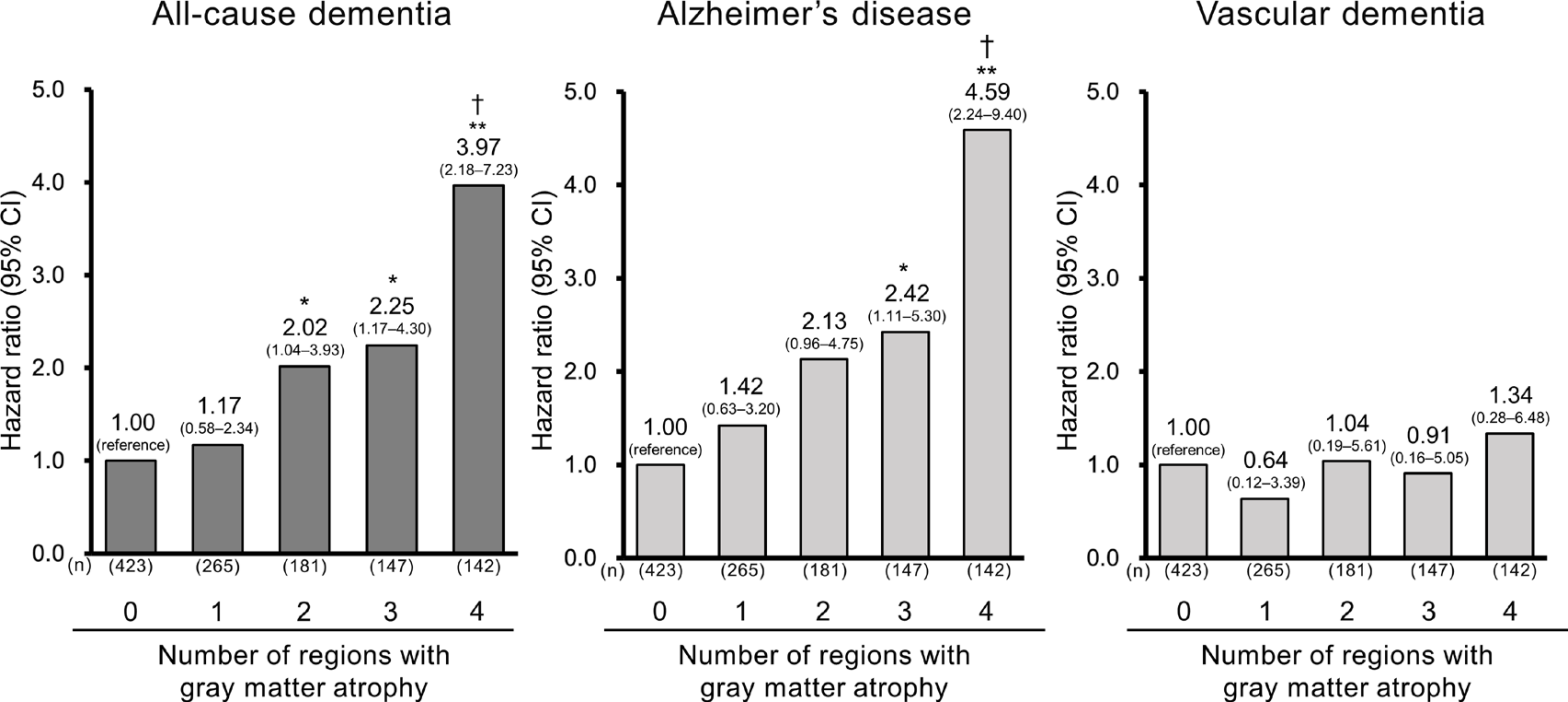
Multivariable-adjusted HR of dementia and its subtypes according to the total number of regions with grey matter atrophy among four dementia-related brain regions. The risk estimates were adjusted for age, sex, education status, systolic blood pressure, use of antihypertensive medication, diabetes mellitus, serum total cholesterol, body mass index, ECG abnormalities, cerebrovascular lesions on MRI, smoking habits, alcohol intake, regular exercise and TBV/ICV. *P<0.05 vs no grey matter atrophy, **p<0.001 vs no grey matter atrophy, †p for trend <0.01. ICV, intracranial volume; TBV, total brain volume.

In addition, we examined the associations between the total number of regions with grey matter atrophy among the four dementia-related brain regions and the risk of all-cause dementia or dementia subtypes. For this analysis, the grey matter atrophy for each of the four brain regions was determined by using ROC curves (online supplemental Table s-7). The risk of all-cause dementia and AD increased gradually with increasing number of regions exhibiting grey matter atrophy (both p for trend <0.001), but the risk of VaD did not (*p* for trend=0.71) (figure 1). Significant associations with the risk of all-cause dementia and AD were observed for subjects with grey matter atrophy in two or more and three or more brain regions, respectively, as compared with those with no grey matter atrophy in all four brain regions.

Finally, we assessed the discrimination and reclassification ability of hippocampal atrophy and the total number of regions exhibiting grey matter atrophy among the four dementia-related brain regions for the development of all-cause dementia and AD (table 6). The model consisting of hippocampal atrophy plus 14 known risk factors for all-cause dementia achieved good discrimination, but not significantly better discrimination (Harrell’s c-statistics: 0.763) than the model consisting of the known risk factors alone (Harrell’s c-statistics: 0.748). On the other hand, when the total number of regions with grey matter atrophy was incorporated into the model consisting of known risk factors, Harrell’s c-statistics of the association with all-cause dementia increased marginally (from 0.748 to 0.775; p=0.08) and that with AD improved significantly (from 0.765 to 0.802, p=0.02). These results further confirmed that the predictive and discriminative ability for the development of all-cause dementia and AD were improved by adding hippocampal atrophy or the total number of regions with grey matter atrophy to the model consisting of known risk factors, respectively.

**Table 6:** Predictive ability, reclassification and discrimination of all-cause dementia and Alzheimer’s disease by hippocampal atrophy and accumulated numbers of grey matter atrophy, 2012–2017

	Harrell’s c-statistics	P value for Harrell’s c-statistics difference (vs basic model)	Continuous NRI (95% CI)	P value for continuous NRI (vs basic model)	IDI (95% CI)	P value for IDI (vs basic model)
All-cause dementia						
Basic model*	0.748	Reference				
Basic model* +hippocampal atrophy	0.763	0.24	0.617 (0.431 to 0.804)	<0.001	0.011 (0.004 to 0.017)	<0.001
Basic model* +accumulated numbers of grey matter atrophy	0.775	0.08	0.508 (0.317 to 0.699)	<0.001	0.020 (0.012 to 0.029)	<0.001
Alzheimer’s disease						
Basic model*	0.765	Reference				
Basic model* +hippocampal atrophy	0.785	0.15	0.610 (0.395 to 0.825)	<0.001	0.011 (0.004 to 0.020)	0.004
Basic model* +accumulated numbers of grey matter atrophy	0.802	0.02	0.465 (0.245 to 0.686)	<0.001	0.022 (0.011 to 0.032)	<0.001

*The basic model included age, sex, education status, systolic blood pressure, antihypertensive medication, diabetes mellitus, serum total cholesterol, body mass index, ECG abnormalities, cerebrovascular lesions on MRI, smoking habits, alcohol intake, regular exercise and the total brain volume-to-intracranial volume ratio.

IDI, integrated discrimination improvement; NRI, net reclassification improvement.

## Discussion

In this prospective study of a general older Japanese population, decreased levels of TBV/ICV and decreased levels of GMV/TBV of the medial temporal lobe, insula, hippocampus and amygdala were significantly associated with a higher risk of dementia. These associations were unchanged when excluding participants with MCI, those with an MMSE score of <24, or those with developing dementia within 1 year of follow-up. In addition, the risks of all-cause dementia and AD increased significantly with higher total number of regions exhibiting grey matter atrophy among four dementia-related brain regions, and the addition of this number into the model consisting of known risk factors improved the predictive ability for developing dementia, especially AD. These findings suggest that the total number of regions with brain atrophy may be an effective neuroimaging biomarker for identifying participants at high-risk for the development of dementia in clinical settings.

Our results showed that decreased levels of GMV/TBV of the medial temporal lobe, insula, hippocampus and amygdala remained closely associated with the risk of dementia even after adjusting for TBV/ICV. Several population-based prospective studies have assessed the association between atrophy in specific brain regions and risk of dementia.^11–15^ The Rotterdam study showed close associations between atrophy of the hippocampus and amygdala and the risk of dementia,^11 12^ and the Three-City Study showed similar associations for the medial temporal lobe, hippocampus, and amygdala.^13 14^ In the Atherosclerosis Risk in Communities (ARIC) Study, lower brain volumes of the hippocampus and the combined brain region consisting of parahippocampal, entorhinal, inferior parietal lobule, precuneus and cuneus were significantly associated with the development of dementia.^15^ On the other hand, no prospective studies have shown a significant association between the insula and risk of dementia. Nonetheless, a few clinical studies reported that participants with MCI and dementia had a lower insula volume than those with normal cognition,^28 29^ and these results support our present findings. Collectively, these previous and our present results suggest that the risk of dementia increases significantly with decreasing volumes of various brain regions associated with core cognitive function.

Neurodegeneration and/or cerebrovascular disease are caused by unfavourable lifestyle habits or lifestyle-related diseases, such as excessive alcohol intake,^30^ hypertension,^31^ diabetes mellitus,^32 33^ stroke,^34^ and traumatic head injury,^35^ in addition to ageing. These risk factors for dementia induce the activation of neuroinflammation and increases in oxidative stress in the brain,^36^ all of which may also promote the deposition of amyloid-β and phosphorylated tau protein.^36–38^ Consequently, the neuronal functional deficit and neuronal cell death due to neurodegeneration and/or cerebrovascular disease could cause the grey matter atrophy of each brain region, and the subsequent progression of cognitive decline.^36 39^ Notably, the medial temporal lobe and hippocampus are known to function primarily in episodic and spatial memory.^40 41^ The insula is reported to be involved in decision making, emotion, and self-cognition, and the sense of taste, smell, and pain.^42^ The amygdala is considered an important region for the formation and storage of both emotional and long-term memories.^43 44^ Therefore, the grey matter atrophy of these brain regions may be involved in the development of dementia.

In this study, the risk of dementia increased significantly with increasing number of brain regions exhibiting grey matter atrophy among four dementia-related brain regions. The assessment of grey matter atrophy in multiple brain regions may be effective for the risk assessment of developing dementia, because it has been reported that different brain regions exhibit grey matter atrophy in different diseases. For example, normal ageing is associated with atrophy of the frontal and temporal lobes,^2–4^ whereas atrophy of the temporal and parietal lobes is characteristic of the development of AD.^1 6–8 11 45^ However, few population-based prospective studies have assessed the association between the number of brain regions exhibiting atrophy and the risk of dementia.^15^ The ARIC study showed that risk of dementia increased significantly with increasing number of AD-related pathological signs, including lobar microhaemorrhages, hippocampus atrophy, and atrophy of the combined brain region consisting of parahippocampal, entorhinal, inferior parietal lobule, precuneus, and cuneus.^15^ Moreover, we demonstrated that the model consisting of the 14 known risk factors for all-cause dementia plus the number of regions exhibiting brain atrophy showed better discrimination ability for developing dementia than either the model consisting of the known risk factors plus hippocampal atrophy or the model consisting of the known risk factors alone. Several prospective studies similarly found that the predictive ability for incident dementia achieved by adding multiple brain regions to a model consisting of potential risk factors for dementia is superior to that achieved by adding hippocampal atrophy to the same basic model or that achieved by the basic model alone.^7 8 15^ These findings support ours. Taken together, these findings suggest that the total number of regions with grey matter atrophy among four dementia-related brain regions, when combined with detailed clinical information, may enable an even more accurate determination of the high-risk population for dementia.

The strengths of our study are the population-based prospective cohort study design, the large sample size of MRI imaging, the consistent and detailed methods of detection and diagnosis of dementia cases in follow-up surveys, the perfect follow-up of participants and the detailed evaluation of known risk factors. However, several limitations of this study should be noted. First, the participants with brain MRI imaging were significantly younger and had significantly higher scores of the MMSE and the Barthel index than those excluded from this study (data not shown). This could have weakened the association found in the current study, biasing the results towards the null hypothesis. Second, there is a possibility that individuals in the prodromal stage of dementia were more likely to be included in participants with lower GMV/TBV at baseline. However, our sensitivity analyses excluding participants with MCI at baseline, participants with an MMSE score of <24, or those who developed dementia within 1 year did not alter any of the results. Third, since we recruited study participants from one town in Japan, the generalisability of the present findings to populations with different genetic backgrounds and lifestyles may be limited. Fourth, we did not collect potential confounding factors that were shown to be risk factors, such as traumatic head injury.

In conclusion, our data showed that decreased values of GMV/TBV in the medial temporal lobe, insula, hippocampus, and amygdala were significantly associated with the development of dementia. In addition, the risk of dementia increased significantly with higher total number of these four dementia-related regions exhibiting grey matter atrophy, and adding this number into the model consisting of known risk factors improved the predictive ability for developing dementia, especially AD. Our findings suggest the need for future researches to target individuals with lower GMV/TBV of dementia-related regions in order to test the reliability of this parameter as a neuroimaging biomarker to assist in the identification of individuals at high risk for dementia in a large population.

## Data Availability

Data are available on reasonable request. Access requires the permission of the Principal Investigator of the Hisayama Study, TNi. The datasets used in the present study are not publicly available because confidential clinical data on the study participants are included.
