# Supplementary material for: Multiple-region grey matter atrophy as a predictor for the development of dementia in a community: the Hisayama Study
Source: J Neurol Neurosurg Psychiatry. 2021 Oct 20;93(3):263–71. doi: 10.1136/jnnp-2021-326611 (PMC8862082; doi:10.1136/jnnp-2021-326611)
Supplement: Supplementary data [file jnnp-2021-326611supp001.pdf]

Supplemental materials

Title: Multiple-region gray matter atrophy as a predictor for the development of dementia in a community: the Hisayama Study

Authors:

Taro Nakazawa, MD[1,2], Tomoyuki Ohara, MD, PhD[1,2], Naoki Hirabayashi, MD, PhD[2,3], Yoshihiko Furuta, MD, PhD[2,4], Jun Hata, MD, PhD[2,4,5], Mao Shibata, MD, PhD[2,3,5], Takanori Honda, PhD[2], Takanari Kitazono, MD, PhD[4,5], Tomohiro Nakao MD, PhD[1], and Toshiharu Ninomiya, MD, PhD[2,5]

Affiliations:

1. Department of Neuropsychiatry, Graduate School of Medical Sciences, Kyushu University, Fukuoka, Japan.
2. Department of Epidemiology and Public Health, Graduate School of Medical Sciences, Kyushu University, Fukuoka, Japan.
3. Department of Psychosomatic Medicine, Graduate School of Medical Sciences, Kyushu University, Fukuoka, Japan.
4. Department of Medicine and Clinical Science, Graduate School of Medical Sciences, Kyushu University, Fukuoka, Japan.
5. Department of Center for Cohort Studies, Graduate School of Medical Sciences, Kyushu University, Fukuoka, Japan.

Corresponding Author:

Dr. Tomoyuki Ohara, M.D., Ph.D.

E-mail address: ohara.tomoyuki.287@m.kyushu-u.ac.jp

Department of Neuropsychiatry, Graduate School of Medical Sciences, Kyushu University

3-1-1 Maidashi, Higashi-ku, Fukuoka 812-8582, Japan

Tel: (+81) 92-642-6151

Fax: (+81) 92-642-4854

SUPPLEMENTAL METHODS

Risk-factor measurements

Each participant answered a self-administered questionnaire that included medical treatment (medications for hypertension, diabetes mellitus, and hypercholesterolemia), medical history, educational status, smoking habits, alcohol intake, and regular exercise. We defined low education as ≤9 years of formal education. We classified smoking habits and alcohol intake as either current habitual or not. Regular exercise was defined as engaging in any form of physical exercise three or more times a week during leisure time. Blood pressure was obtained 3 times using an automated sphygmomanometer after more than 5 min rest in the sitting position, and we used the mean of 3 measurements for the analysis. Hypertension was defined as blood pressure levels ≥140/90 mmHg and/or current use of antihypertensive medication. Plasma glucose levels were determined by the hexokinase method. Diabetes mellitus was defined as follows: fasting glucose level ≥7.0 mmol/L, casual or 2-h postload glucose levels ≥11.1 mmol/L and/or use of antidiabetic medications. Serum total cholesterol levels were determined enzymatically. Body mass index (BMI) was calculated with body weight and height, which were measured in light clothing without shoes. Electrocardiogram abnormalities were defined by ST depression (Minnesota Code. 4-1, 2, 3), left ventricular hypertrophy (3-1), or atrial fibrillation (8-3).

MRI analysis

Using a 1.5-Tesla MRI scanner (Intera Pulsar; Philips Medical Systems, Best, the Netherlands) with a multichannel head coil, we examined 3-dimensional T1-weighted images, conventional T1- and T2-weighted images, fluid attenuated inversion recovery (FLAIR), T2*-weighted images, and magnetic resonance angiographic images of the brain. The 3-dimensional T1-weighted images were converted to Neuroimaging Informatics

Technology Initiative format and then segmented into three components (gray matter, white matter, and cerebrospinal fluid) by using VBM8 Toolbox version 435 (University of Jena, Germany) in SPM8 (University College London, UK) running in MATLAB (The Mathworks, Inc, Natick, MA). The International Consortium for Brain Mapping template for East Asian brains was used as an anatomical setting. Since white matter hyperintensities (WMH) were often misclassified as gray matter, we corrected white matter and gray matter images by using binarized WMH masks. Segmented gray matter images were normalized and modulated to compensate for the volumetric effects of expansion/shrinking in spatial normalization. Based on the preceding cortical parcellation, the gray matter volume (GMV) of the frontal, temporal, medial temporal (including entorhinal and parahippocampus), parietal, occipital, and insular lobes and the cingulate, hippocampus, accumbens, amygdala, caudate, pallidum, putamen, and thalamus were computed using the Neuromorphometrics atlas of SPM12 (Neuromorphometrics, Somerville, MA). The remaining gray matter voxels were labeled according to the nearest cortical label with a 5-mm distance limit. The total brain volume (TBV) was calculated as the sum of the gray matter and white matter volumes. The intracranial volume (ICV) was calculated as the sum of the TBV and the cerebrospinal fluid volumes. We calculated the TBV to ICV ratio (TBV/ICV) as an indicator of global brain atrophy. As an indicator of regional gray matter atrophy beyond total brain atrophy, the GMV to TBV ratio (GMV/TBV) for each brain region was calculated.

Cerebrovascular lesions were defined as brain infarction or hemorrhage on MRI regardless of the presence or absence of neurological symptoms. Brain infarction was defined as lesions of ≥3 mm in diameter visible on both the T1-weighted image (as a hypointense lesion) and the T2-weighted image (as a hyperintense lesion) with a surrounding hyperintense rim on the FLAIR image. Brain hemorrhage was defined as any hemorrhagic lesions, including cerebral microbleeds, visible on the T2*-weighted image (as a hypointense lesion). Each MRI scan

was checked by two trained stroke physicians blinded to the clinical information (inter-rater agreement ratio: 74.8% for the brain infarctions, 83.7% for the brain hemorrhages). In the case of conflicting interpretations, a third stroke physician checked the scan and made the final determination.

Statistical analysis

We tested trends of the age- and sex-adjusted mean values and frequencies of risk factors across the quartiles of the total brain volume to intracranial volume ratio (TBV/ICV) by using a linear or logistic regression analysis, respectively. We used the Spearman correlation coefficient to assess the correlations between each risk factor and either the gray matter volume to TBV ratio (GMV/TBV) in each brain region or the TBV/ICV. The hazard ratios (HRs) and their 95% confidence intervals of the quartiles of TBV/ICV or the quartiles of GMV/TBV in each brain region for the development of dementia and its subtypes were computed by using a Cox proportional hazards model. We evaluated three different models: (1) model 1, adjusted for age and sex; (2) model 2 adjusted for age, sex, education status, systolic blood pressure, antihypertensive medications, diabetes mellitus, serum total cholesterol, body mass index, electrocardiogram abnormalities, cerebrovascular lesions on MRI, smoking habits, alcohol intake, and regular exercise; and (3) model 3, adjusted for the covariates included in model 2 plus TBV/ICV. In the sensitivity analysis, we excluded those who had MCI, those with cognitive decline defined as an MMSE score <24,[1] or those who developed dementia within one year. The linear trends in the risk of dementia across each quartile of TBV/ICV or each quartile of GMV/TBV in each brain region were tested by assigning an ordinal number (i.e., 1, 2, 3, or 4) to each quartile. Regarding the multiple comparisons of the association between GMV/TBV in each brain region and risk of dementia among the 14 selected brain regions, false discovery rate (FDR) correction[2] was performed to

verify the multiple comparisons for which a significance level with a q-value of FDR correction was defined as <0.10.[3]

We also assessed the association between the total number of regions exhibiting gray matter atrophy among four dementia-related brain regions and the risk of dementia. For this purpose, we first estimated the maximum likelihood point on the receiver operating characteristic (ROC) curve[4] to determine the cut-off value of the presence and absence of GMV/TBV atrophy for each of the four brain regions associated with dementia, and then calculated the total number of the regions with gray matter atrophy. We compared the accuracy of risk assessment for the development of all-cause dementia and AD between the models including known dementia risk factors with and without hippocampal atrophy or the total number of dementia-related brain regions with gray matter atrophy. The predictive ability of each model was assessed by the Harrell's c-statistics, and the increase in the Harrell's c-statistics between models was evaluated by using a method reported in 2010.[5] The increased predictive ability of the model including hippocampal atrophy or the total number of dementia-related brain regions with gray matter atrophy was further examined by using the continuous net reclassification improvement (NRI) and integrated discrimination improvement (IDI),[6] where the individual probabilities were estimated by using the relevant Cox model. We carried out all the statistical analyses using SAS software version 9.4 (SAS institute, Cary, NC) and Stata version 14.0 (Stata Corp, College Station, TX). Statistical significance was defined as a two-sided value of $p < 0.05$.

References (for the supplemental method)

1. Tsoi KKF, Chan JYC, Hirai HW, et al. Cognitive tests to detect dementia: a systematic review and meta-analysis. *Jama Intern Med* 2015;175:1450–1458.
2. Benjamini, Y., & Hochberg, Y. Controlling the False Discovery Rate: A Practical and Powerful Approach to Multiple Testing. *J. R. Statist. Soc. ser.B* 1995;57:298–300
3. Genovese CR, Lazar NA, Nichols T. Thresholding of Statistical Maps in Functional Neuroimaging Using the False Discovery Rate. *NeuroImage* 2002;15:870–878
4. Perkins NJ, Schisterman EF. The inconsistency of “optimal” cutpoints obtained using two criteria based on the receiver operating characteristic curve. *Am J Epidemiol* 2006;163:670–675.
5. Newson RB. Comparing the predictive powers of survival models using Harrell’s C or Somers’ D. *Stata J* 2010;10:339–358.
6. Pencina MJ, D’Agostino RB, Steyerberg EW. Extensions of net reclassification improvement calculations to measure usefulness of new biomarkers. *Stat Med* 2011;30:11–21.

Table s–1. Age– and sex–adjusted Spearman's correlation coefficients of the TBV/ICV or GMV/TBV ratios of each brain lobe with potential risk factors for dementia at baseline

Risk factors at baseline	TBV/ICV	Frontal GMV/TBV	Temporal GMV/TBV	Medial Temporal GMV/TBV	Parietal GMV/TBV	Occipital GMV/TBV	Insular GMV/TBV
Age, years	-0.57*	-0.38*	-0.37*	-0.15*	-0.21*	-0.14*	-0.21*
Male sex, %	-0.31*	-0.25*	-0.05	-0.06*	-0.31*	-0.02	-0.21*
Education ≤ 9 years, %	0.03	0.05	-0.03	-0.02	-0.01	0.06	0.02
Systolic blood pressure, mmHg	-0.01	-0.02	-0.05	0.01	-0.01	-0.01	-0.01
Diastolic blood pressure, mmHg	0.04	-0.02	-0.04	-0.01	-0.01	0.01	0.01
Antihypertensive medication, %	-0.07*	-0.04	-0.06*	0.04	-0.04	-0.08*	0.01
Hypertension, %	-0.06*	-0.03	-0.06	0.04	-0.03	-0.08*	0.03
Diabetes mellitus, %	-0.15*	-0.02	-0.07*	-0.01	0.003	-0.01	-0.03
Serum total cholesterol, mmol/L	0.06	-0.01	0.03	-0.01	0.02	-0.02	0.02
Body mass index, kg/m^2	0.01	-0.04	-0.11*	0.01	-0.03	-0.05	0.003
Electrocardiogram abnormalities, %	-0.03	0.01	-0.02	-0.03	0.02	-0.01	0.02
Cerebrovascular lesions, %	-0.05	-0.07*	-0.06*	0.01	-0.01	0.01	0.01
Smoking habits, %	-0.06*	0.02	0.02	-0.04	0.06	0.13*	-0.02
Alcohol intake, %	-0.03	-0.01	-0.01	-0.01	0.01	0.02	-0.01
Regular exercise, %	-0.004	0.001	0.02	0.01	0.0002	0.03	-0.02
MMSE < 24, %	-0.03	0.02	-0.02	-0.03	0.03	-0.01	-0.01
Mild cognitive impairment, %	-0.05	-0.04	-0.04	-0.03	0.01	0.02	-0.05

Abbreviations: TBV, total brain volume; ICV, intracranial volume; GMV, gray matter volume; SD, standard deviation; MMSE, Mini–Mental Statement Examination.

Age was adjusted for sex. Sex was adjusted for age.

Hypertension was defined as blood pressure ≥ 140/90mmHg and/or current use of antihypertensive medication. Electrocardiogram abnormalities were defined as Minnesota Code 3-1, 4-1, 4-2, 4-3 or 8-3. Regular exercise was defined as engaging in any forms of physical exercise three or more times a week during leisure time. Cerebrovascular lesions were defined as brain infarction of hemorrhage on MRI regardless of the presence of absence of neurological symptoms. Smoking habits and alcohol intake were classified as current use or not.

* $p < 0.05$

Table s-2. Age– and sex–adjusted Spearman's correlation coefficients of GMV to TBV ratios of basal ganglia, limbic system, and thalamus with potential risk factors for dementia at baseline

Risk factors at baseline	Cingulate GMV/TBV	Hippocampal GMV/TBV	Accumbens GMV/TBV	Amygdala GMV/TBV	Caudate GMV/TBV	Pallidum GMV/TBV	Putamen GMV/TBV	Thalamus GMV/TBV
Age, years	-0.22*	-0.31*	-0.46*	-0.35*	-0.44*	-0.02*	-0.20*	-0.32*
Male sex, %	-0.21*	-0.25*	-0.18*	-0.10*	-0.27*	-0.11*	-0.19*	-0.24*
Education ≤ 9 years, %	0.01	-0.001	0.02	0.001	0.05	-0.03	0.04	-0.002
Systolic blood pressure, mmHg	-0.01	0.01	0.02	0.03	-0.002	0.06	0.08*	0.004
Diastolic blood pressure, mmHg	-0.01	0.02	0.02	0.04	-0.02	0.05	0.08*	0.05
Antihypertensive medication, %	-0.04	0.06	-0.02	0.04	-0.03	0.02	0.05	-0.01
Hypertension, %	-0.03	0.05	-0.01	0.05	-0.01	0.04	0.09*	0.02
Diabetes mellitus, %	0.04	-0.03	-0.06*	-0.01	-0.09*	0.01	-0.03	-0.09*
Serum total cholesterol, mmol/L	-0.01	0.02	0.01	0.01	0.05	0.01	0.01	0.05
Body mass index, kg/m^2	-0.01	0.03	0.01	0.05	-0.01	-0.03	0.005	-0.08*
Electrocardiogram abnormalities, %	-0.05	-0.01	-0.003	0.02	-0.004	0.05	0.05	0.04
Cerebrovascular lesions, %	-0.03	0.03	-0.05	0.001	-0.07*	0.02	0.10*	-0.004
Smoking habits, %	0.01	0.04	-0.02	-0.03	-0.05	-0.001	-0.05	-0.02
Alcohol intake, %	-0.03	-0.02	-0.04	0.003	0.02	0.07*	-0.004	-0.01
Regular exercise, %	-0.01	0.04	0.01	0.005	-0.003	0.02	-0.02	-0.02
MMSE < 24, %	-0.003	-0.05	-0.02	-0.07*	0.02	0.001	-0.002	-0.03
Mild cognitive impairment, %	-0.06*	-0.05	-0.09*	-0.07*	-0.05	-0.02	-0.08*	-0.05

Abbreviations: TBV, total brain volume; ICV, intracranial volume; GMV, gray matter volume; SD, standard deviation; MMSE, Mini–Mental Statement Examination.

Age was adjusted for sex. Sex was adjusted for age.

Hypertension was defined as blood pressure ≥ 140/90mmHg and/or current use of antihypertensive medication. Electrocardiogram abnormalities were defined as Minnesota Code 3-1, 4-1, 4-2, 4-3 or 8-3. Regular exercise was defined as engaging in any forms of physical exercise three or more times a week during leisure time. Cerebrovascular lesions were defined as brain infarction of hemorrhage on MRI regardless of the presence of absence of neurological symptoms. Smoking habits and alcohol intake were classified as current use or not.

* $p < 0.05$

Table s-3. The multivariable adjusted *p* for trend and q-value of FDR correction for the association between quartiles of the gray matter volume-to-total brain volume ratio in each brain region and risk of all-cause dementia.

	p for trend	*q*- value of FDR correction
Hippocampal GMV/TBV (%)	<.001	0.001
Amygdala GMV/TBV (%)	0.003	0.02
Insular GMV/TBV (%)	0.004	0.02
Medial Temporal GMV/TBV (%)	0.02	0.06
Parietal GMV/TBV (%)	0.06	0.18
Accumbens GMV/TBV (%)	0.08	0.18
Temporal GMV/TBV (%)	0.14	0.27
Cingulate GMV/TBV (%)	0.36	0.63
Occipital GMV/TBV (%)	0.43	0.67
Thalamus GMV/TBV (%)	0.46	0.67
Putamen GMV/TBV (%)	0.63	0.80
Caudate GMV/TBV (%)	0.93	1.00
Frontal GMV/TBV (%)	0.96	1.00
Pallidum GMV/TBV (%)	0.99	1.00

Abbreviations: TBV, total brain volume; ICV, intracranial volume; GMV, gray matter volume

Multivariate adjustment was made for age, sex, education status, systolic blood pressure, antihypertensive medication, diabetes mellitus, serum total cholesterol, body mass index, electrocardiogram abnormalities, cerebrovascular lesions on MRI, smoking habits, alcohol intake, regular exercise, and TBV/ICV.

Table s-4. Multivariable–adjusted hazard ratios (95% CI) of all-cause dementia according to quartiles of total brain volume to intracranial volume ratio and gray matter volume to total brain volume ratio of medial temporal lobe, insula, hippocampus, and amygdala, after excluding 157 subjects with mild cognitive impairment at baseline

	No. of subjects	No. of events	Multivariable–adjusted hazard ratio (95% CI)
TBV/ICV (%)			
Q1 (71.54–76.81)	234	31	2.94 (1.11–7.82)* ¶
Q2 (76.82–78.43)	240	21	2.05 (0.79–5.38) ¶
Q3 (78.44–79.80)	254	17	2.45 (0.94–6.34) ¶
Q4 (79.81–85.48)	273	6	1.00 (Reference)
			p for trend = 0.06
Medial Temporal GMV/TBV (%)			
Q1 (0.40–0.79)	242	37	2.50 (1.28–4.87)*
Q2 (0.80–0.84)	247	13	1.12 (0.51–2.47)
Q3 (0.85–0.89)	260	13	1.10 (0.49–2.49)
Q4 (0.90–1.09)	252	12	1.00 (Reference)
			p for trend = 0.003
Insular GMV/TBV (%)			
Q1 (0.61–1.03)	238	36	2.63 (1.29–5.34)*
Q2 (1.04–1.10)	251	16	1.40 (0.63–3.07)
Q3 (1.11–1.17)	253	11	0.81 (0.35–1.88)
Q4 (1.18–1.46)	259	12	1.00 (Reference)
			p for trend < 0.001
Hippocampal GMV/TBV (%)			
Q1 (0.45–0.77)	237	39	3.53 (1.55–8.00)*
Q2 (0.78–0.81)	248	17	1.83 (0.77–4.37)
Q3 (0.82–0.86)	255	11	1.13 (0.44–2.89)
Q4 (0.87–1.01)	261	8	1.00 (Reference)
			p for trend < 0.001
Amygdala GMV/TBV (%)			
Q1 (0.11–0.21)	227	37	2.18 (1.06–4.50)*
Q2 (0.22–0.23)	257	20	1.63 (0.77–3.45)
Q3 (0.24–0.24)	253	7	0.55 (0.20–1.50)
Q4 (0.25–0.31)	264	11	1.00 (Reference)
			p for trend = 0.004

Abbreviations: TBV, total brain volume; ICV, intracranial volume; GMV, gray matter volume

Multivariate adjustment was made for age, sex, education status, systolic blood pressure, antihypertensive medication, diabetes mellitus, serum total cholesterol, body mass index, electrocardiogram abnormalities, cerebrovascular lesions on MRI, smoking habits, alcohol intake, regular exercise, and TBV/ICV.

¶Adjusted for all variables in the multivariate adjustment except for TBV/ICV.

Table s-5. Multivariable–adjusted hazard ratios (95% CI) of all-cause dementia according to quartiles of the total brain volume-to-intracranial volume ratio and gray matter volume-to-total brain volume ratio of the medial temporal lobe, insula, hippocampus, and amygdala, after excluding subjects with an MMSE score <24 at baseline

	No. of subjects	No. of events	Multivariable–adjusted hazard ratio (95% CI)
TBV/ICV (%)			
Q1 (71.54–76.81)	265	45	4.90 (1.80–13.36)* ¶
Q2 (76.82–78.43)	271	28	3.25 (1.20–8.78)* ¶
Q3 (78.44–79.80)	277	18	2.97 (1.08–8.14)* ¶
Q4 (79.81–85.48)	279	5	1.00 (Reference)
			p for trend = 0.002
Medial Temporal GMV/TBV (%)			
Q1 (0.40–0.79)	271	43	1.69 (0.98–2.91)
Q2 (0.80–0.84)	269	16	0.82 (0.41–1.59)
Q3 (0.85–0.89)	276	17	0.89 (0.46–1.74)
Q4 (0.90–1.09)	276	20	1.00 (Reference)
			p for trend = 0.04
Insular GMV/TBV (%)			
Q1 (0.61–1.03)	270	44	1.74 (0.95–3.19)
Q2 (1.04–1.10)	275	22	1.07 (0.55–2.08)
Q3 (1.11–1.17)	273	13	0.59 (0.28–1.24)
Q4 (1.18–1.46)	274	17	1.00 (Reference)
			p for trend = 0.01
Hippocampal GMV/TBV (%)			
Q1 (0.45–0.77)	265	47	2.49 (1.23–5.06)*
Q2 (0.78–0.81)	274	23	1.67 (0.79–3.52)
Q3 (0.82–0.86)	275	15	1.05 (0.47–2.34)
Q4 (0.87–1.01)	278	11	1.00 (Reference)
			p for trend = 0.001
Amygdala GMV/TBV (%)			
Q1 (0.11–0.21)	259	48	1.82 (0.96–3.43)
Q2 (0.22–0.23)	278	21	1.32 (0.67–2.63)
Q3 (0.24–0.24)	279	13	0.80 (0.37–1.75)
Q4 (0.25–0.31)	276	14	1.00 (Reference)
			p for trend = 0.02

Abbreviations: TBV, total brain volume; ICV, intracranial volume; GMV, gray matter volume

Multivariate adjustment was made for age, sex, education status, systolic blood pressure, antihypertensive medication, diabetes mellitus, serum total cholesterol, body mass index, electrocardiogram abnormalities, cerebrovascular lesions on MRI, smoking habits, alcohol intake, regular exercise, and TBV/ICV.

¶Adjusted for all variables in the multivariate adjustment except for TBV/ICV.

Table s-6. Multivariable–adjusted hazard ratios (95% CI) of all-cause dementia according to quartiles of the total brain volume-to-intracranial volume ratio and gray matter volume-to-total brain volume ratio of the medial temporal lobe, insula, hippocampus, and amygdala, after censoring subjects who developed all-cause dementia during the initial one year of follow-up

	No. of subjects	No. of events	Multivariable–adjusted hazard ratio (95% CI)
TBV/ICV (%)			
Q1 (71.54–76.81)	277	47	3.24 (1.35–7.76)* ¶
Q2 (76.82–78.43)	287	31	2.33 (0.99–5.53) ¶
Q3 (78.44–79.80)	290	21	2.34 (0.98–5.62) ¶
Q4 (79.81–85.48)	288	7	1.00 (Reference)
			p for trend = 0.01
Medial Temporal GMV/TBV (%)			
Q1 (0.40–0.79)	279	47	1.78 (1.04–3.05)*
Q2 (0.80–0.84)	290	19	0.92 (0.48–1.73)
Q3 (0.85–0.89)	287	20	1.01 (0.53–1.92)
Q4 (0.90–1.09)	286	20	1.00 (Reference)
			p for trend = 0.02
Insular GMV/TBV (%)			
Q1 (0.61–1.03)	280	47	1.77 (0.99–3.18)
Q2 (1.04–1.10)	284	24	1.11 (0.59–2.11)
Q3 (1.11–1.17)	290	17	0.75 (0.38–1.48)
Q4 (1.18–1.46)	288	18	1.00 (Reference)
			p for trend = 0.01
Hippocampal GMV/TBV (%)			
Q1 (0.45–0.77)	279	53	3.31 (1.61–6.81)*
Q2 (0.78–0.81)	287	26	2.19 (1.04–4.64)*
Q3 (0.82–0.86)	288	17	1.39 (0.62–3.09)
Q4 (0.87–1.01)	288	10	1.00 (Reference)
			p for trend < .001
Amygdala GMV/TBV (%)			
Q1 (0.11–0.21)	279	54	2.18 (1.14–4.16)*
Q2 (0.22–0.23)	288	24	1.54 (0.78–3.06)
Q3 (0.24–0.24)	288	15	0.97 (0.45–2.08)
Q4 (0.25–0.31)	287	13	1.00 (Reference)
			p for trend = 0.003

Abbreviations: TBV, total brain volume; ICV, intracranial volume; GMV, gray matter volume

Multivariate adjustment was made for age, sex, education status, systolic blood pressure, antihypertensive medication, diabetes mellitus, serum total cholesterol, body mass index, electrocardiogram abnormalities, cerebrovascular lesions on MRI, smoking habits, alcohol intake, regular exercise, and TBV/ICV.

¶Adjusted for all variables in the multivariate adjustment except for TBV/ICV.

Table s-7. Cut-off values, C-statistics, sensitivity, and specificity of each dementia-related brain region based on the maximum likelihood point on the receiver operating characteristic curves

	Cut-off value	C-statistics	Sensitivity (%)	Specificity (%)
Medial Temporal GMV/TBV (%)	0.819	0.618	58.4	65.5
Insular GMV/TBV (%)	1.076	0.641	63.7	64.7
Hippocampal GMV/TBV (%)	0.798	0.688	64.6	63.6
Amygdala GMV/TBV (%)	0.220	0.690	57.5	74.0

Abbreviations: TBV, total brain volume; GMV, gray matter volume
